# Generating confidence intervals on biological networks

**DOI:** 10.1186/1471-2105-8-467

**Published:** 2007-11-30

**Authors:** Thomas Thorne, Michael PH Stumpf

**Affiliations:** 1 Division of Molecular Biosciences, Imperial College London, Wolfson Building, London SW7 2AZ, UK; 2 Institute of Mathematical Sciences, Imperial College London, London, UK

## Abstract

**Background:**

In the analysis of networks we frequently require the statistical significance of some network statistic, such as measures of similarity for the properties of interacting nodes. The structure of the network may introduce dependencies among the nodes and it will in general be necessary to account for these dependencies in the statistical analysis. To this end we require some form of Null model of the network: generally rewired replicates of the network are generated which preserve only the degree (number of interactions) of each node. We show that this can fail to capture important features of network structure, and may result in unrealistic significance levels, when potentially confounding additional information is available.

**Methods:**

We present a new network resampling Null model which takes into account the degree sequence as well as available biological annotations. Using gene ontology information as an illustration we show how this information can be accounted for in the resampling approach, and the impact such information has on the assessment of statistical significance of correlations and motif-abundances in the *Saccharomyces cerevisiae *protein interaction network. An algorithm, GOcardShuffle, is introduced to allow for the efficient construction of an improved Null model for network data.

**Results:**

We use the protein interaction network of *S. cerevisiae*; correlations between the evolutionary rates and expression levels of interacting proteins and their statistical significance were assessed for Null models which condition on different aspects of the available data. The novel GOcardShuffle approach results in a Null model for annotated network data which appears better to describe the properties of real biological networks.

**Conclusion:**

An improved statistical approach for the statistical analysis of biological network data, which conditions on the available biological information, leads to qualitatively different results compared to approaches which ignore such annotations. In particular we demonstrate the effects of the biological organization of the network can be sufficient to explain the observed similarity of interacting proteins.

## Background

Large-scale protein interaction network (PIN) data have now been collected in a number of prokaryotic and eukaryotic species. It has been suggested that these networks provide an integrative perspective on cellular processes and considerable effort has been invested into their functional and evolutionary analysis[1-6]. At the moment molecular network data sets are still plagued by noise [7] – this is especially true for protein interaction networks – and incompleteness [8], but nevertheless considerable progress is being made in the analysis of complex cellular phenotypes in light of such networks. Below we will introduce an novel method for the construction of confidence intervals for network quantities. This new approach is able to fuse different lines of biological information and generate conditional confidence intervals; these can be applied as an alternative we can employ it in addition to demonstrate it in an analysis of the *Saccaromyces cerevisiae *PIN.

A number of studies have investigated (i) whether characteristics of interacting proteins are more similar than those of proteins for which no interaction has been reported [9,10], and (ii) how the network structure affects properties – such as the evolutionary rate [6,11-13] – of interacting genes or proteins. Other studies have looked at structural properties, such as network motifs [14-16]. Because of the dependence introduced by the network it is, however, not possible to use the conventional confidence measures, *e.g. *for the correlation coefficient of some property of pairs of interacting proteins. Rather, a network-aware Null model has to be used which compares the actual network with some suitably randomized version of it. In order to incorporate network aspects these studies have used either (i) straightforward bootstrapping of nodes in order to create random pairs of nodes (such as proteins) [9,10], (ii) bootstrapped nodes based on their degree [6], or (iii) randomly rewired the network while keeping the degree of each node fixed [14,15,17] (see Methods section for details). The first approach has been shown to underestimate the size of the confidence intervals (CI) [6], while the second and third yield statistically similar results (CIs are also broader than for (i)) for measures of pair-wise similarity of the properties of interacting nodes. In order to assess the CIs for motifs, however, an explicit incorporation of the network is generally necessary and only the third approach can be used.

All of these three approaches above rely, however, implicitly on the assumption that the network is uniform and not structured in any particular way. Such procedures also ignore any other information that is increasingly becoming available for many species [18-20], and which may affect the organization of the network. While bootstrap (as long as the degree sequence is accounted for either exactly or statistically) or rewiring approaches are parsimonious – and undoubtedly should be preferred for general cases where no other information is available – it opens up the question as to whether such approaches are still satisfactory when additional, potentially co-variate, data is available.

Here we provide statistical tools for incorporating such additional information into the statistical analysis. Using such information can have considerable effect on the construction of network confidence intervals, and our procedure, GOcardShuffle, constructs randomly rewired instances of networks that incorporate the degree sequence exactly, and additional information statistically (based on a rejection-sampling algorithm). Thus, for example, if in a given protein interaction dataset, proteins in the mitochondria interact predominantly with other mitochondrial proteins but not at all with proteins in the endoplasmic reticulum, then GOcardShuffle will construct only instances of randomly rewired networks which reflect the relative importance of intra-category versus inter-category connections. In addition to GO annotations, any other biological annotation (e.g *Enzyme Commission *numbers or protein domain information) may act as confounding variables, *e.g. *when expression levels differ between categories.

There is a rich statistical literature on confounding variables and their role in the statistical interpretation of primary effects. Scenarios, where the effects of known or unknown confounding variables result in inconsistencies unless properly accounted for, are known as examples of Simpson's paradox in statistics. On a much more subtle scale there will undoubtedly be confounding variables in many of the processes studied in systems biology. These, at least in principle, can be accounted for in a framework such as GOcardShuffle; if the approach (implemented in Python and R) is used in addition to random rewiring then it may be possible to detect such potentially confounding hidden variables.

## Results

Here we illustrate the use of GOcardShuffle by contrasting statistical confidence intervals obtained under different Null models for network rewiring.

### Correlation of node properties

**Figure 1:**
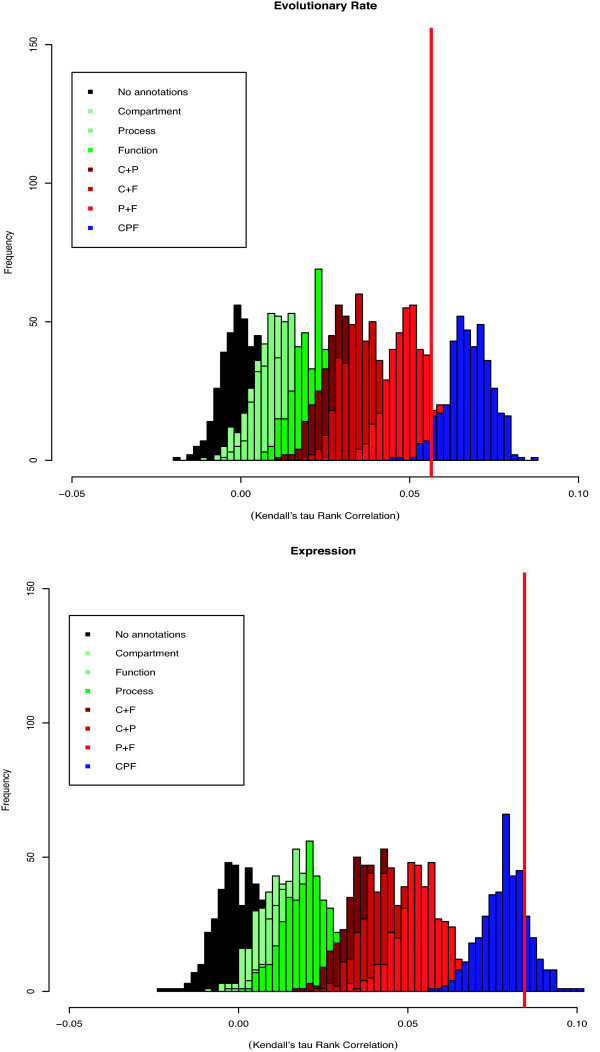
**Confidence intervals generated from GOcardShuffle**. Confidence intervals for the correlation of evolutionary rates and expression levels (mRNA expression levels are used as proxies for protein abundance). Incorporating GO annotations, individually, in pairs, or all three categories together results in progressive right-shifts of the distribution under the conditional Null models. The real data is indicated by the red vertical lines. When all three categories of biological information are included the distribution obtained under the Null model covers the observed correlation for both evolutionary rates and expression levels of interacting proteins. Function, Process and Compartment are indicated by F, P and C, respectively (the approximation Eqn. (12) was used to calculate the weight matrices for multiple annotation categories; for the yeast dataset used here this appears to be a reasonable approximation).

Figure 1 shows the correlations between the evolutionary rates of interacting proteins, and between expression levels of interacting proteins (observed values are indicated by vertical red lines) in the *S. cerevisiae *PIN. Correlation is measured using Kendall's *τ *rank correlation statistic (other correlation measures can be used and are available in the software implementation of GOcardShuffle; the Pearson and Spearman correlation measures result in qualitatively identical results to those shown here). The histograms show the distributions resulting from 1000 independently rewired networks using no annotation (black), one category (red hues), two categories (green hues) and all three categories (blue) simultaneously. While the most parsimonious Null model (black) results in a distribution which is centered around *τ *= 0, including annotation in the rewiring procedure (via GOcardShuffle) leads to a systematic shift towards positive values of *τ*. Interestingly, the shift experienced depends on the GO category in different ways for the correlation of evolutionary rates and that of expression levels; this reflects presumably the effect different categories have on evolutionary rate and expression level measures respectively: annotations related to "function" appear to have a greater effect in explaining correlations among the expression values of interacting proteins, whereas the "process" annotation has a greater impact on the expected correlations of expression levels of interacting proteins. The "cellular component" annotation appears to have the least important impact. This is in agreement with the results of Agrafioti *et al*. who found more significant differences in the evolutionary rates among proteins with different functions than processes [6].

In both cases, however, we notice that the full annotation as used in GOcardShuffle results in distributions of correlation values that cover the observed value of the correlation. Thus, once the rewired network instances are conditioned on GO annotations the observed correlation appear to be covered by the new, conditioned Null model. In Figure 1 in Additional File 1 we show that the effects of conditioning on presently available functional information in the context of presently available protein interaction data does result in a shift of the distribution obtained under the Null model away from zero to finite positive values. Depending on the dataset and correlation measure, however, the GOcardShuffle histogram may not overlap the observed value (see Additional File 1).

Quite generally, we expect that conditioning such analyses on additional available data (of which increasing amounts are becoming available) will result in a shift in the expected Null distribution if such data does explain some aspects of the variability in the measures to be correlated. That is, we observe the shift in the Null distributions, precisely because some of the variation in evolutionary rate and mRNA expression levels are captured by GO annotations [6,21].

### Network motifs

**Figure 2:**
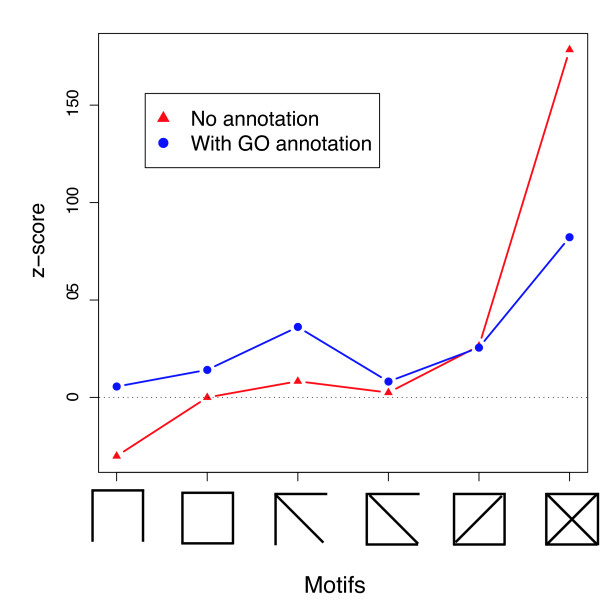
**Significance of motifs evaluated using GOcardShuffle**. Significance, evaluated by *z*-scores, of motifs also changes when annotation is included in the statistical analysis. We note that now all motifs of four nodes are over-represented in the true network, compared to the ensemble of conditionally randomized networks; interestingly the over-representation of the completely connected graph with 4 nodes (measured by its *z*-score) is halved once annotation has been taken into account.

In order to illustrate the use of GOcardShuffle on motif-analysis [14,15] we counted the numbers of each possible motif of size four present in the original protein interaction network (as captured by DIP, see *Methods *section) and in each of the rewired networks. The statistical significance of motifs is assessed by their *Z *scores (see *Methods*). For the simple null model, and the GOcardShuffle Null model using all three annotation categories, these are shown in Figure 2. Changing the Null model against which significance is assessed naturally changes the observed *Z*-scores of the motifs. Perhaps the most interesting result is that the relative excess of the fully connected motif in the true network compared to the "random networks" decreases as the annotations are taken into account. Another way of looking at this is that incorporating the coarse structure of the PIN (as captured to some extent at least by GO data) cannot account for the local network patterns across the network.

## Discussion

We have shown that it is possible to condition the rewiring process by which confidence intervals on networks are constructed on biological information such as gene ontology data. Integrating such known biological information into the statistical analysis of protein interaction network data may result in changes to the Null model if such data is correlated with network organization. We demonstrated the effect of conditioning on GO data by analyzing the correlations among interacting proteins: several studies had reported that properties of interacting proteins are significantly more similar than those of non-interacting proteins. Applying GOcardShuffle to yeast PIN data and conditioning on different combinations of GO categories suggests that this may at least partially be because the protein interaction networks of real biological organisms are inhomogeneous and show a level of local and functional organization, which has been ignored in previous statistical analyses. In light of the conditional Null models, however, the similarity of evolutionary rates and expression levels of interacting proteins in the Yeast PIN dataset used here, is just as would be expected for a network with the same biological characteristics (as captured by present biological annotations). Since these protein characteristics differ between different categories [6] – even if sometimes only slightly – and since within-category interactions are more frequent than between category interactions, similarity of properties of interacting proteins are readily understood.

Presently GO annotations have to be treated with some care and caution. There is the danger of circular arguments if *in-silico *annotations (which often rely on protein-protein interactions) are used. As we outline in the *Methods *section uncertainties and different levels of support for different annotations are straightforwardly incorporated into the GOcardShuffle algorithm.

The source code of the Python and R routines of the implementation of GOcardShuffle are available from the authors' website [22].

## Conclusion

Our novel network resampling approach allows the construction of confidence intervals under a statistical Null model of network organization which conditions on available biological information. If used in addition to conventional rewiring procedures then this approach can be used to detect potentially confounding hidden variables or relationships in systems biology data.

GOcardShuffle allows the refinement of the statistical Null model for network structure based on available biological data: the rewired network instances may now capture probabilistically the modular aspects of these molecular networks (if the annotations imply such a structure). This appears to be the case for GO annotations of yeast proteins, and as we have shown, such stratification of the network – where within-category interactions are more frequent than between-category interactions – may lead to correlations among properties of interacting proteins. Once this has been accounted for, there is no strong additional evidence for interacting proteins to be more similar than would be expected by chance. The present approach is readily extended to include other information on functional and structural properties of the network. Quite generally, GOcardShuffle, can be applied in the statistical analysis of coloured graph problems.

## Methods

### Data

**Figure 3:**
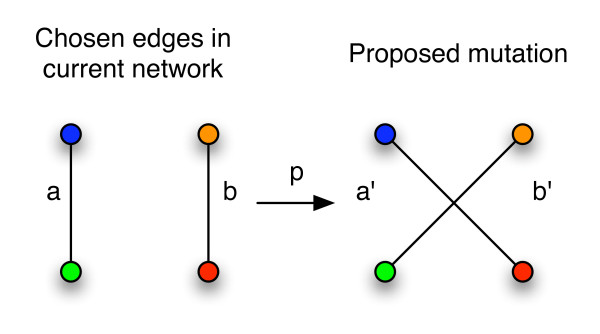
**Updating of network configuration**. The configuration change (*a*, *b*) → (*a'*, *b'*) is accepted according to Eqn. (6).

In the illustrative examples (Figures 1, 2, 3) protein interaction data was taken from the Database of Interacting Proteins [23,24]; other databases contain similar information [25,26] (and the effect of GOcardShuffle is the same for these datasets). GO annotations in a flat-file format can be obtained from the *Saccaromyces Genome Database *[18]; similar lists of GO annotations, rather than the hierarchical structures can be generated by a number of programs and tools such as FatiGO [27,28]. Evolutionary information was taken from the study of Agrafioti *et al*. [6]. The mRNA expression data of Cho *et al*. [29] was taken as a proxy for protein abundance.

### Constructing confidence intervals for networks

Given a reported network dataset (which will at present generally be plagued by false-positive and false-negative results [7,30], as well as incompleteness [31]) we wish to be able to evaluate the statistical significance of some network statistic. To this end we need to construct networks which share some characteristics of the observed network; as we have shown above, the choice of the information we choose to use to generate such rewired networks can have a pronounced effect on the results of the statistical analysis.

#### Previous approaches: unconditional procedures

Depending on whether the similarity of properties of interacting proteins or the abundance of network motifs were considered, previous approaches assessed statistical significance either through a bootstrap or randomization procedure, or by rewiring the network. In the former authors either picked *M *pairs of interacting proteins by randomly sampling 2*M *proteins with replacement from the *N *proteins in the dataset [9], shuffled the list of interaction partners [10], or picked proteins proportional to their degree [6]. The latter two approaches conserve the degree sequence exactly and probabilistically, respectively; for the first approach, on the other hand, it is straightforward to show, that this corresponds to making the assumption that the Null model is a classical or Erdös-Rényi random graph (and is therefore inappropriate for the analysis of real networks). The sample statistic (such as a correlation coefficient) is then calculated for each replicate to generate the distribution under the Null model.

Rewiring of the network involves breaking up all interactions and leaving a number of "stubs" at each node corresponding to its degree. Randomly chosen pairs of stubs are then connected until all *M *interactions have been created and the summary statistic (correlation coefficient or number of motifs) is calculated. Repeating this process a sufficient number of times again results in the expected distribution under the Null model. Furthermore a Markov Chain Monte Carlo approach can be constructed which, *e.g. *conditions the network on the number of observed triangles [14]. Such an approach is in practice, however, computationally expensive and does not appear to have been used widely [15]. In the meantime, however, elegant analytic approaches have been developed which allow the statistical assessment of network motif exceptionality [32].

#### Conditional rewiring: GOcardShuffle

To include biological knowledge and potential co-variates, such as GO annotations, in the resampling process the method given in the algorithm below is used. Let *N *and *M *be the number of nodes and edges in the network, respectively; let *γ *be the set of annotations (*e.g. *different protein functions), and let *γ*(*i*) be the annotation of node *i*. For *x*, *y *∈ *γ *we define *ν*_*xy *_to be the number of edges that connect a node with annotation *x *to a node with annotation *y*. Then the probability of picking a random stub on a node with annotation *x *that has an edge attached that leads to a node with annotation *y *(we say that the edge is of type (*x*, *y*)) is given by

ωxy=νxy2Mfor x≠y
 MathType@MTEF@5@5@+=feaafiart1ev1aaatCvAUfKttLearuWrP9MDH5MBPbIqV92AaeXatLxBI9gBaebbnrfifHhDYfgasaacPC6xNi=xI8qiVKYPFjYdHaVhbbf9v8qqaqFr0xc9vqFj0dXdbba91qpepeI8k8fiI+fsY=rqGqVepae9pg0db9vqaiVgFr0xfr=xfr=xc9adbaqaaeGacaGaaiaabeqaaeqabiWaaaGcbaqbaeqabeGaaaqaaGGaciab=L8a3naaBaaaleaacqWG4baEcqWG5bqEaeqaaOGaeyypa0tcfa4aaSaaaeaacqWF9oGBdaWgaaqaaiabdIha4jabdMha5bqabaaabaGaeGOmaiJaemyta0eaaaGcbaGaeeOzayMaee4Ba8MaeeOCaiNaeeiiaaIaemiEaGNaeyiyIKRaemyEaKhaaaaa@435C@

and

ωxx=νxxMotherwise.
 MathType@MTEF@5@5@+=feaafiart1ev1aaatCvAUfKttLearuWrP9MDH5MBPbIqV92AaeXatLxBI9gBaebbnrfifHhDYfgasaacPC6xNi=xI8qiVKYPFjYdHaVhbbf9v8qqaqFr0xc9vqFj0dXdbba91qpepeI8k8fiI+fsY=rqGqVepae9pg0db9vqaiVgFr0xfr=xfr=xc9adbaqaaeGacaGaaiaabeqaaeqabiWaaaGcbaqbaeqabeGaaaqaaGGaciab=L8a3naaBaaaleaacqWG4baEcqWG4baEaeqaaOGaeyypa0tcfa4aaSaaaeaacqWF9oGBdaWgaaqaaiabdIha4jabdIha4bqabaaabaGaemyta0eaaaGcbaGaee4Ba8MaeeiDaqNaeeiAaGMaeeyzauMaeeOCaiNaee4DaCNaeeyAaKMaee4CamNaeeyzauMaeiOla4caaaaa@4618@

This definition means that the probabilities are properly normalized, *i.e. *∑*ω*_*xy *_= 1, where the sum runs over all pairs of indices 1 ≤ *x*, *y *≤ |*γ*^*k*^|. If #*x *denotes the number of *x*, then normalization follows from the relationship

1M(12#edges of type(x,y)+12#edges of type(y,x)+#edges of type(x,x))=∑x≠yωxy+∑xωxx=1
 MathType@MTEF@5@5@+=feaafiart1ev1aaatCvAUfKttLearuWrP9MDH5MBPbIqV92AaeXatLxBI9gBaebbnrfifHhDYfgasaacPC6xNi=xI8qiVKYPFjYdHaVhbbf9v8qqaqFr0xc9vqFj0dXdbba91qpepeI8k8fiI+fsY=rqGqVepae9pg0db9vqaiVgFr0xfr=xfr=xc9adbaqaaeGacaGaaiaabeqaaeqabiWaaaGcbaqcfa4aaSaaaeaacqaIXaqmaeaacqWGnbqtaaGcdaqadaqaaKqbaoaalaaabaGaeGymaedabaGaeGOmaidaaOGaei4iamIaeeyzauMaeeizaqMaee4zaCMaeeyzauMaee4CamNaeeiiaaIaee4Ba8MaeeOzayMaeeiiaaIaeeiDaqNaeeyEaKNaeeiCaaNaeeyzauMaeiikaGIaemiEaGNaeiilaWIaemyEaKNaeiykaKIaey4kaSscfa4aaSaaaeaacqaIXaqmaeaacqaIYaGmaaGccqGGJaWicqqGLbqzcqqGKbazcqqGNbWzcqqGLbqzcqqGZbWCcqqGGaaicqqGVbWBcqqGMbGzcqqGGaaicqqG0baDcqqG5bqEcqqGWbaCcqqGLbqzcqGGOaakcqWG5bqEcqGGSaalcqWG4baEcqGGPaqkcqGHRaWkcqGGJaWicqqGLbqzcqqGKbazcqqGNbWzcqqGLbqzcqqGZbWCcqqGGaaicqqGVbWBcqqGMbGzcqqGGaaicqqG0baDcqqG5bqEcqqGWbaCcqqGLbqzcqGGOaakcqWG4baEcqGGSaalcqWG4baEcqGGPaqkaiaawIcacaGLPaaacqGH9aqpdaaeqbqaaGGaciab=L8a3naaBaaaleaacqWG4baEcqWG5bqEaeqaaaqaaiabdIha4jabgcMi5kabdMha5bqab0GaeyyeIuoakiabgUcaRmaaqafabaGae8xYdC3aaSbaaSqaaiabdIha4jabdIha4bqabaaabaGaemiEaGhabeqdcqGHris5aOGaeyypa0JaeGymaedaaa@9432@

because the first sum on the RHS of Eqn. (3) runs over all ordered pairs of distinct annotations *x *and *y*. We approximate the likelihood of a given network N
 MathType@MTEF@5@5@+=feaafiart1ev1aaatCvAUfKttLearuWrP9MDH5MBPbIqV92AaeXatLxBI9gBaebbnrfifHhDYfgasaacPC6xNi=xH8viVGI8Gi=hEeeu0xXdbba9frFj0xb9qqpG0dXdb9aspeI8k8fiI+fsY=rqGqVepae9pg0db9vqaiVgFr0xfr=xfr=xc9adbaqaaeGacaGaaiaabeqaaeqabiWaaaGcbaWenfgDOvwBHrxAJfwnHbqeg0uy0HwzTfgDPnwy1aaceaGae8xdX7eaaa@3761@ = (V
 MathType@MTEF@5@5@+=feaafiart1ev1aaatCvAUfKttLearuWrP9MDH5MBPbIqV92AaeXatLxBI9gBaebbnrfifHhDYfgasaacPC6xNi=xH8viVGI8Gi=hEeeu0xXdbba9frFj0xb9qqpG0dXdb9aspeI8k8fiI+fsY=rqGqVepae9pg0db9vqaiVgFr0xfr=xfr=xc9adbaqaaeGacaGaaiaabeqaaeqabiWaaaGcbaWenfgDOvwBHrxAJfwnHbqeg0uy0HwzTfgDPnwy1aaceaGae8xfXBfaaa@3771@, ℰ
 MathType@MTEF@5@5@+=feaafiart1ev1aaatCvAUfKttLearuWrP9MDH5MBPbIqV92AaeXatLxBI9gBaebbnrfifHhDYfgasaacPC6xNi=xH8viVGI8Gi=hEeeu0xXdbba9frFj0xb9qqpG0dXdb9aspeI8k8fiI+fsY=rqGqVepae9pg0db9vqaiVgFr0xfr=xfr=xc9adbaqaaeGacaGaaiaabeqaaeqabiWaaaGcbaWenfgDOvwBHrxAJfwnHbqeg0uy0HwzTfgDPnwy1aaceaGae8hmHueaaa@36AB@) (where V
 MathType@MTEF@5@5@+=feaafiart1ev1aaatCvAUfKttLearuWrP9MDH5MBPbIqV92AaeXatLxBI9gBaebbnrfifHhDYfgasaacPC6xNi=xH8viVGI8Gi=hEeeu0xXdbba9frFj0xb9qqpG0dXdb9aspeI8k8fiI+fsY=rqGqVepae9pg0db9vqaiVgFr0xfr=xfr=xc9adbaqaaeGacaGaaiaabeqaaeqabiWaaaGcbaWenfgDOvwBHrxAJfwnHbqeg0uy0HwzTfgDPnwy1aaceaGae8xfXBfaaa@3771@ and ℰ
 MathType@MTEF@5@5@+=feaafiart1ev1aaatCvAUfKttLearuWrP9MDH5MBPbIqV92AaeXatLxBI9gBaebbnrfifHhDYfgasaacPC6xNi=xH8viVGI8Gi=hEeeu0xXdbba9frFj0xb9qqpG0dXdb9aspeI8k8fiI+fsY=rqGqVepae9pg0db9vqaiVgFr0xfr=xfr=xc9adbaqaaeGacaGaaiaabeqaaeqabiWaaaGcbaWenfgDOvwBHrxAJfwnHbqeg0uy0HwzTfgDPnwy1aaceaGae8hmHueaaa@36AB@ denote the sets of nodes and edges, respectively) as the product of the probability of edges conditional on the annotations of the nodes incident on the edge. The probability of an edge, *e*(*i*, *j*) between two nodes with annotations *γ*(*i*) and *γ*(*j*) is given by *ω*_*e *_:= *ω*_*γ*(*i*)*γ*(*j*) _whence we approximate Pr(N
 MathType@MTEF@5@5@+=feaafiart1ev1aaatCvAUfKttLearuWrP9MDH5MBPbIqV92AaeXatLxBI9gBaebbnrfifHhDYfgasaacPC6xNi=xH8viVGI8Gi=hEeeu0xXdbba9frFj0xb9qqpG0dXdb9aspeI8k8fiI+fsY=rqGqVepae9pg0db9vqaiVgFr0xfr=xfr=xc9adbaqaaeGacaGaaiaabeqaaeqabiWaaaGcbaWenfgDOvwBHrxAJfwnHbqeg0uy0HwzTfgDPnwy1aaceaGae8xdX7eaaa@3761@) ≈ Pr(ℰ
 MathType@MTEF@5@5@+=feaafiart1ev1aaatCvAUfKttLearuWrP9MDH5MBPbIqV92AaeXatLxBI9gBaebbnrfifHhDYfgasaacPC6xNi=xH8viVGI8Gi=hEeeu0xXdbba9frFj0xb9qqpG0dXdb9aspeI8k8fiI+fsY=rqGqVepae9pg0db9vqaiVgFr0xfr=xfr=xc9adbaqaaeGacaGaaiaabeqaaeqabiWaaaGcbaWenfgDOvwBHrxAJfwnHbqeg0uy0HwzTfgDPnwy1aaceaGae8hmHueaaa@36AB@) and we have thus for our likelihood of the network

ℒ(N)=Pr⁡(ω|N)≈∏e∈ℰωe
 MathType@MTEF@5@5@+=feaafiart1ev1aaatCvAUfKttLearuWrP9MDH5MBPbIqV92AaeXatLxBI9gBaebbnrfifHhDYfgasaacPC6xNi=xI8qiVKYPFjYdHaVhbbf9v8qqaqFr0xc9vqFj0dXdbba91qpepeI8k8fiI+fsY=rqGqVepae9pg0db9vqaiVgFr0xfr=xfr=xc9adbaqaaeGacaGaaiaabeqaaeqabiWaaaGcbaWenfgDOvwBHrxAJfwnHbqeg0uy0HwzTfgDPnwy1aaceaGae8NeHWKaeiikaGIae8xdX7KaeiykaKIaeyypa0JagiiuaaLaeiOCaiNaeiikaGccciGae4xYdCNaeiiFaWNae8xdX7KaeiykaKIaeyisIS7aaebuaeaacqGFjpWDdaWgaaWcbaGaemyzaugabeaaaeaacqWGLbqzcqGHiiIZcqWFWesraeqaniabg+Givdaaaa@4FC7@

Given a configuration, N
 MathType@MTEF@5@5@+=feaafiart1ev1aaatCvAUfKttLearuWrP9MDH5MBPbIqV92AaeXatLxBI9gBaebbnrfifHhDYfgasaacPC6xNi=xH8viVGI8Gi=hEeeu0xXdbba9frFj0xb9qqpG0dXdb9aspeI8k8fiI+fsY=rqGqVepae9pg0db9vqaiVgFr0xfr=xfr=xc9adbaqaaeGacaGaaiaabeqaaeqabiWaaaGcbaWenfgDOvwBHrxAJfwnHbqeg0uy0HwzTfgDPnwy1aaceaGae8xdX7eaaa@3761@ = (V
 MathType@MTEF@5@5@+=feaafiart1ev1aaatCvAUfKttLearuWrP9MDH5MBPbIqV92AaeXatLxBI9gBaebbnrfifHhDYfgasaacPC6xNi=xH8viVGI8Gi=hEeeu0xXdbba9frFj0xb9qqpG0dXdb9aspeI8k8fiI+fsY=rqGqVepae9pg0db9vqaiVgFr0xfr=xfr=xc9adbaqaaeGacaGaaiaabeqaaeqabiWaaaGcbaWenfgDOvwBHrxAJfwnHbqeg0uy0HwzTfgDPnwy1aaceaGae8xfXBfaaa@3771@, ℰ
 MathType@MTEF@5@5@+=feaafiart1ev1aaatCvAUfKttLearuWrP9MDH5MBPbIqV92AaeXatLxBI9gBaebbnrfifHhDYfgasaacPC6xNi=xH8viVGI8Gi=hEeeu0xXdbba9frFj0xb9qqpG0dXdb9aspeI8k8fiI+fsY=rqGqVepae9pg0db9vqaiVgFr0xfr=xfr=xc9adbaqaaeGacaGaaiaabeqaaeqabiWaaaGcbaWenfgDOvwBHrxAJfwnHbqeg0uy0HwzTfgDPnwy1aaceaGae8hmHueaaa@36AB@) we propose a novel configuration N
 MathType@MTEF@5@5@+=feaafiart1ev1aaatCvAUfKttLearuWrP9MDH5MBPbIqV92AaeXatLxBI9gBaebbnrfifHhDYfgasaacPC6xNi=xH8viVGI8Gi=hEeeu0xXdbba9frFj0xb9qqpG0dXdb9aspeI8k8fiI+fsY=rqGqVepae9pg0db9vqaiVgFr0xfr=xfr=xc9adbaqaaeGacaGaaiaabeqaaeqabiWaaaGcbaWenfgDOvwBHrxAJfwnHbqeg0uy0HwzTfgDPnwy1aaceaGae8xdX7eaaa@3761@*' *= (V
 MathType@MTEF@5@5@+=feaafiart1ev1aaatCvAUfKttLearuWrP9MDH5MBPbIqV92AaeXatLxBI9gBaebbnrfifHhDYfgasaacPC6xNi=xH8viVGI8Gi=hEeeu0xXdbba9frFj0xb9qqpG0dXdb9aspeI8k8fiI+fsY=rqGqVepae9pg0db9vqaiVgFr0xfr=xfr=xc9adbaqaaeGacaGaaiaabeqaaeqabiWaaaGcbaWenfgDOvwBHrxAJfwnHbqeg0uy0HwzTfgDPnwy1aaceaGae8xfXBfaaa@3771@, ℰ
 MathType@MTEF@5@5@+=feaafiart1ev1aaatCvAUfKttLearuWrP9MDH5MBPbIqV92AaeXatLxBI9gBaebbnrfifHhDYfgasaacPC6xNi=xH8viVGI8Gi=hEeeu0xXdbba9frFj0xb9qqpG0dXdb9aspeI8k8fiI+fsY=rqGqVepae9pg0db9vqaiVgFr0xfr=xfr=xc9adbaqaaeGacaGaaiaabeqaaeqabiWaaaGcbaWenfgDOvwBHrxAJfwnHbqeg0uy0HwzTfgDPnwy1aaceaGae8hmHueaaa@36AB@*'*) (the set of nodes does not change hence N
 MathType@MTEF@5@5@+=feaafiart1ev1aaatCvAUfKttLearuWrP9MDH5MBPbIqV92AaeXatLxBI9gBaebbnrfifHhDYfgasaacPC6xNi=xH8viVGI8Gi=hEeeu0xXdbba9frFj0xb9qqpG0dXdb9aspeI8k8fiI+fsY=rqGqVepae9pg0db9vqaiVgFr0xfr=xfr=xc9adbaqaaeGacaGaaiaabeqaaeqabiWaaaGcbaWenfgDOvwBHrxAJfwnHbqeg0uy0HwzTfgDPnwy1aaceaGae8xdX7eaaa@3761@*' *= N
 MathType@MTEF@5@5@+=feaafiart1ev1aaatCvAUfKttLearuWrP9MDH5MBPbIqV92AaeXatLxBI9gBaebbnrfifHhDYfgasaacPC6xNi=xH8viVGI8Gi=hEeeu0xXdbba9frFj0xb9qqpG0dXdb9aspeI8k8fiI+fsY=rqGqVepae9pg0db9vqaiVgFr0xfr=xfr=xc9adbaqaaeGacaGaaiaabeqaaeqabiWaaaGcbaWenfgDOvwBHrxAJfwnHbqeg0uy0HwzTfgDPnwy1aaceaGae8xdX7eaaa@3761@) by choosing two edges, *e*, *f *∈ ℰ
 MathType@MTEF@5@5@+=feaafiart1ev1aaatCvAUfKttLearuWrP9MDH5MBPbIqV92AaeXatLxBI9gBaebbnrfifHhDYfgasaacPC6xNi=xH8viVGI8Gi=hEeeu0xXdbba9frFj0xb9qqpG0dXdb9aspeI8k8fiI+fsY=rqGqVepae9pg0db9vqaiVgFr0xfr=xfr=xc9adbaqaaeGacaGaaiaabeqaaeqabiWaaaGcbaWenfgDOvwBHrxAJfwnHbqeg0uy0HwzTfgDPnwy1aaceaGae8hmHueaaa@36AB@, at random. We consider the ordered tuple of their annotations (*u*, *v*) and (*x*, *y*), respectively and propose new edges by swapping the edges between the nodes (see Figure 3) to obtain edges *e' *and *f' *which will be of type (*x*, *v*) and (*u*, *y*), respectively. The likelihood ratio is thus

ℒ(N′)ℒ(N)=∏e∈ℰ′ωe∏e∈ℰωe=ωe′ωf′ωeωf,
MathType@MTEF@5@5@+=feaafiart1ev1aaatCvAUfKttLearuWrP9MDH5MBPbIqV92AaeXatLxBI9gBaebbnrfifHhDYfgasaacPC6xNi=xI8qiVKYPFjYdHaVhbbf9v8qqaqFr0xc9vqFj0dXdbba91qpepeI8k8fiI+fsY=rqGqVepae9pg0db9vqaiVgFr0xfr=xfr=xc9adbaqaaeGacaGaaiaabeqaaeqabiWaaaGcbaWaaSaaaeaat0uy0HwzTfgDPnwy1egaryqtHrhAL1wy0L2yHvdaiqaacqWFsectcqGGOaakcuWFneVtgaqbaiabcMcaPaqaaiab=jrimjabcIcaOiab=1q8ojabcMcaPaaacqGH9aqpdaWcaaqaamaarababaacciGae4xYdC3aaSbaaSqaaiabdwgaLbqabaaabaGaemyzauMaeyicI4Saf8hmHuKbauaaaeqaniabg+Givdaakeaadaqeqaqaaiab+L8a3naaBaaaleaacqWGLbqzaeqaaaqaaiabdwgaLjabgIGiolab=btifbqab0Gaey4dIunaaaGccqGH9aqpjuaGdaWcaaqaaiab+L8a3naaBaaabaGafmyzauMbauaaaeqaaiab+L8a3naaBaaabaGafmOzayMbauaaaeqaaaqaaiab+L8a3naaBaaabaGaemyzaugabeaacqGFjpWDdaWgaaqaaiabdAgaMbqabaaaaOGaeiilaWcaaa@61DE@

as all other edges in ℰ
 MathType@MTEF@5@5@+=feaafiart1ev1aaatCvAUfKttLearuWrP9MDH5MBPbIqV92AaeXatLxBI9gBaebbnrfifHhDYfgasaacPC6xNi=xH8viVGI8Gi=hEeeu0xXdbba9frFj0xb9qqpG0dXdb9aspeI8k8fiI+fsY=rqGqVepae9pg0db9vqaiVgFr0xfr=xfr=xc9adbaqaaeGacaGaaiaabeqaaeqabiWaaaGcbaWenfgDOvwBHrxAJfwnHbqeg0uy0HwzTfgDPnwy1aaceaGae8hmHueaaa@36AB@ and ℰ
 MathType@MTEF@5@5@+=feaafiart1ev1aaatCvAUfKttLearuWrP9MDH5MBPbIqV92AaeXatLxBI9gBaebbnrfifHhDYfgasaacPC6xNi=xH8viVGI8Gi=hEeeu0xXdbba9frFj0xb9qqpG0dXdb9aspeI8k8fiI+fsY=rqGqVepae9pg0db9vqaiVgFr0xfr=xfr=xc9adbaqaaeGacaGaaiaabeqaaeqabiWaaaGcbaWenfgDOvwBHrxAJfwnHbqeg0uy0HwzTfgDPnwy1aaceaGae8hmHueaaa@36AB@*' *remain unaffected by the proposed change.

We start from a random rewiring of the network which only conserves the degree of each node. The rewiring algorithm is based on Markov Chain Monte Carlo (MCMC) approach using Metropolis sampling [33,34], and begins with a randomly rewired network with the desired degree sequence (see Additional File 1). A pair of edges *e *= (*i*, *j*), *f *= (*r*, *s*) is chosen randomly and the incident nodes are found to have annotations *γ*(*i*), *γ*(*j*) and *γ*(*r*), *γ*(*s*), respectively, in the *κ *different categories. Then the probability of the original and the rewired networks differ only by the weights of the involved edges. The probability of accepting the new configuration, *e' *= (*i*, *s*), *f' *= (*j*, *r*) is thus given by the Metropolis criterion

p=h(N,N′)=min⁡(1,ℒ(N′)ℒ(N))=min⁡(1,ω′eω′fωeωf)
 MathType@MTEF@5@5@+=feaafiart1ev1aaatCvAUfKttLearuWrP9MDH5MBPbIqV92AaeXatLxBI9gBaebbnrfifHhDYfgasaacPC6xNi=xI8qiVKYPFjYdHaVhbbf9v8qqaqFr0xc9vqFj0dXdbba91qpepeI8k8fiI+fsY=rqGqVepae9pg0db9vqaiVgFr0xfr=xfr=xc9adbaqaaeGacaGaaiaabeqaaeqabiWaaaGcbaGaemiCaaNaeyypa0JaemiAaGMaeiikaGYenfgDOvwBHrxAJfwnHbqeg0uy0HwzTfgDPnwy1aaceaGae8xdX7KaeiilaWIaf8xdX7KbauaacqGGPaqkcqGH9aqpcyGGTbqBcqGGPbqAcqGGUbGBdaqadaqaaiabigdaXiabcYcaSmaalaaabaGae8NeHWKaeiikaGIaf8xdX7KbauaacqGGPaqkaeaacqWFsectcqGGOaakcqWFneVtcqGGPaqkaaaacaGLOaGaayzkaaGaeyypa0JagiyBa0MaeiyAaKMaeiOBa42aaeWaaeaacqaIXaqmcqGGSaaljuaGdaWcaaqaaGGaciqb+L8a3zaafaWaaSbaaeaacqWGLbqzaeqaaiqb+L8a3zaafaWaaSbaaeaacqWGMbGzaeqaaaqaaiab+L8a3naaBaaabaGaemyzaugabeaacqGFjpWDdaWgaaqaaiabdAgaMbqabaaaaaGccaGLOaGaayzkaaaaaa@67CE@

The configuration remains unchanged with probability 1 - *p*, whence a new configuration change will be proposed.

It is easy to see that the ensemble of networks which condition on the observed edge weights, *ω*, form the stationary distribution of the Markov chain thus constructed. To show this we let *r*(N
 MathType@MTEF@5@5@+=feaafiart1ev1aaatCvAUfKttLearuWrP9MDH5MBPbIqV92AaeXatLxBI9gBaebbnrfifHhDYfgasaacPC6xNi=xH8viVGI8Gi=hEeeu0xXdbba9frFj0xb9qqpG0dXdb9aspeI8k8fiI+fsY=rqGqVepae9pg0db9vqaiVgFr0xfr=xfr=xc9adbaqaaeGacaGaaiaabeqaaeqabiWaaaGcbaWenfgDOvwBHrxAJfwnHbqeg0uy0HwzTfgDPnwy1aaceaGae8xdX7eaaa@3761@ → N
 MathType@MTEF@5@5@+=feaafiart1ev1aaatCvAUfKttLearuWrP9MDH5MBPbIqV92AaeXatLxBI9gBaebbnrfifHhDYfgasaacPC6xNi=xH8viVGI8Gi=hEeeu0xXdbba9frFj0xb9qqpG0dXdb9aspeI8k8fiI+fsY=rqGqVepae9pg0db9vqaiVgFr0xfr=xfr=xc9adbaqaaeGacaGaaiaabeqaaeqabiWaaaGcbaWenfgDOvwBHrxAJfwnHbqeg0uy0HwzTfgDPnwy1aaceaGae8xdX7eaaa@3761@*'*) be the transition mechanism of the chain,

r(N→N′)=q(N→N′)×h(N,N′)
 MathType@MTEF@5@5@+=feaafiart1ev1aaatCvAUfKttLearuWrP9MDH5MBPbIqV92AaeXatLxBI9gBaebbnrfifHhDYfgasaacPC6xNi=xI8qiVKYPFjYdHaVhbbf9v8qqaqFr0xc9vqFj0dXdbba91qpepeI8k8fiI+fsY=rqGqVepae9pg0db9vqaiVgFr0xfr=xfr=xc9adbaqaaeGacaGaaiaabeqaaeqabiWaaaGcbaGaemOCaiNaeiikaGYenfgDOvwBHrxAJfwnHbqeg0uy0HwzTfgDPnwy1aaceaGae8xdX7KaeyOKH4Qaf8xdX7KbauaacqGGPaqkcqGH9aqpcqWGXbqCcqGGOaakcqWFneVtcqGHsgIRcuWFneVtgaqbaiabcMcaPiabgEna0kabdIgaOjabcIcaOiab=1q8ojabcYcaSiqb=1q8ozaafaGaeiykaKcaaa@5206@

where *q*(N
 MathType@MTEF@5@5@+=feaafiart1ev1aaatCvAUfKttLearuWrP9MDH5MBPbIqV92AaeXatLxBI9gBaebbnrfifHhDYfgasaacPC6xNi=xH8viVGI8Gi=hEeeu0xXdbba9frFj0xb9qqpG0dXdb9aspeI8k8fiI+fsY=rqGqVepae9pg0db9vqaiVgFr0xfr=xfr=xc9adbaqaaeGacaGaaiaabeqaaeqabiWaaaGcbaWenfgDOvwBHrxAJfwnHbqeg0uy0HwzTfgDPnwy1aaceaGae8xdX7eaaa@3761@ → N
 MathType@MTEF@5@5@+=feaafiart1ev1aaatCvAUfKttLearuWrP9MDH5MBPbIqV92AaeXatLxBI9gBaebbnrfifHhDYfgasaacPC6xNi=xH8viVGI8Gi=hEeeu0xXdbba9frFj0xb9qqpG0dXdb9aspeI8k8fiI+fsY=rqGqVepae9pg0db9vqaiVgFr0xfr=xfr=xc9adbaqaaeGacaGaaiaabeqaaeqabiWaaaGcbaWenfgDOvwBHrxAJfwnHbqeg0uy0HwzTfgDPnwy1aaceaGae8xdX7eaaa@3761@*'*) is the probability of going from network N
 MathType@MTEF@5@5@+=feaafiart1ev1aaatCvAUfKttLearuWrP9MDH5MBPbIqV92AaeXatLxBI9gBaebbnrfifHhDYfgasaacPC6xNi=xH8viVGI8Gi=hEeeu0xXdbba9frFj0xb9qqpG0dXdb9aspeI8k8fiI+fsY=rqGqVepae9pg0db9vqaiVgFr0xfr=xfr=xc9adbaqaaeGacaGaaiaabeqaaeqabiWaaaGcbaWenfgDOvwBHrxAJfwnHbqeg0uy0HwzTfgDPnwy1aaceaGae8xdX7eaaa@3761@ to N
 MathType@MTEF@5@5@+=feaafiart1ev1aaatCvAUfKttLearuWrP9MDH5MBPbIqV92AaeXatLxBI9gBaebbnrfifHhDYfgasaacPC6xNi=xH8viVGI8Gi=hEeeu0xXdbba9frFj0xb9qqpG0dXdb9aspeI8k8fiI+fsY=rqGqVepae9pg0db9vqaiVgFr0xfr=xfr=xc9adbaqaaeGacaGaaiaabeqaaeqabiWaaaGcbaWenfgDOvwBHrxAJfwnHbqeg0uy0HwzTfgDPnwy1aaceaGae8xdX7eaaa@3761@*'*. Here this step will always involve swapping of two edges. These, however, are chosen uniformly at random and therefore

q(N′→N)=q(N→N′)
 MathType@MTEF@5@5@+=feaafiart1ev1aaatCvAUfKttLearuWrP9MDH5MBPbIqV92AaeXatLxBI9gBaebbnrfifHhDYfgasaacPC6xNi=xI8qiVKYPFjYdHaVhbbf9v8qqaqFr0xc9vqFj0dXdbba91qpepeI8k8fiI+fsY=rqGqVepae9pg0db9vqaiVgFr0xfr=xfr=xc9adbaqaaeGacaGaaiaabeqaaeqabiWaaaGcbaGaemyCaeNaeiikaGYenfgDOvwBHrxAJfwnHbqeg0uy0HwzTfgDPnwy1aaceaGaf8xdX7KbauaacqGHsgIRcqWFneVtcqGGPaqkcqGH9aqpcqWGXbqCcqGGOaakcqWFneVtcqGHsgIRcuWFneVtgaqbaiabcMcaPaaa@4854@

With this it is trivial to show that the detailed balance [35] is fulfilled, *i.e.*

L(N)r(N→N′)=L(N)q(N→N′)h(N,N′)=L(N)q(N→N′)min⁡(1,ℒ(N′)ℒ(N))=q(N→N′)min⁡(ℒ(N),ℒ(N′))=L(N′)q(N′→N)h(N′,N)=L(N′)r(N′→N).
 MathType@MTEF@5@5@+=feaafiart1ev1aaatCvAUfKttLearuWrP9MDH5MBPbIqV92AaeXatLxBI9gBaebbnrfifHhDYfgasaacPC6xNi=xI8qiVKYPFjYdHaVhbbf9v8qqaqFr0xc9vqFj0dXdbba91qpepeI8k8fiI+fsY=rqGqVepae9pg0db9vqaiVgFr0xfr=xfr=xc9adbaqaaeGacaGaaiaabeqaaeqabiWaaaGcbaqbaeaabqWaaaaabaGaemitaWKaeiikaGYenfgDOvwBHrxAJfwnHbqeg0uy0HwzTfgDPnwy1aaceaGae8xdX7KaeiykaKIaemOCaiNaeiikaGIae8xdX7KaeyOKH4Qaf8xdX7KbauaacqGGPaqkaeaacqGH9aqpaeaacqWGmbatcqGGOaakcqWFneVtcqGGPaqkcqWGXbqCcqGGOaakcqWFneVtcqGHsgIRcuWFneVtgaqbaiabcMcaPiabdIgaOjabcIcaOiab=1q8ojabcYcaSiqb=1q8ozaafaGaeiykaKcabaaabaGaeyypa0dabaGaemitaWKaeiikaGIae8xdX7KaeiykaKIaemyCaeNaeiikaGIae8xdX7KaeyOKH4Qaf8xdX7KbauaacqGGPaqkcyGGTbqBcqGGPbqAcqGGUbGBdaqadaqaaiabigdaXiabcYcaSmaalaaabaGae8NeHWKaeiikaGIaf8xdX7KbauaacqGGPaqkaeaacqWFsectcqGGOaakcqWFneVtcqGGPaqkaaaacaGLOaGaayzkaaaabaaabaGaeyypa0dabaGaemyCaeNaeiikaGIae8xdX7KaeyOKH4Qaf8xdX7KbauaacqGGPaqkcyGGTbqBcqGGPbqAcqGGUbGBcqGGOaakcqWFsectcqGGOaakcqWFneVtcqGGPaqkcqGGSaalcqWFsectcqGGOaakcuWFneVtgaqbaiabcMcaPiabcMcaPaqaaaqaaiabg2da9aqaaiabdYeamjabcIcaOiqb=1q8ozaafaGaeiykaKIaemyCaeNaeiikaGIaf8xdX7KbauaacqGHsgIRcqWFneVtcqGGPaqkcqWGObaAcqGGOaakcuWFneVtgaqbaiabcYcaSiab=1q8ojabcMcaPiabg2da9iabdYeamjabcIcaOiqb=1q8ozaafaGaeiykaKIaemOCaiNaeiikaGIaf8xdX7KbauaacqGHsgIRcqWFneVtcqGGPaqkcqGGUaGlaaaaaa@B73F@

Thus GOcardShuffle – because of the general properties of MCMC [34,35] – will result in a Markov chain which has as its stationary distribution the ensemble of networks (defined by Pr(*ω*|N
 MathType@MTEF@5@5@+=feaafiart1ev1aaatCvAUfKttLearuWrP9MDH5MBPbIqV92AaeXatLxBI9gBaebbnrfifHhDYfgasaacPC6xNi=xH8viVGI8Gi=hEeeu0xXdbba9frFj0xb9qqpG0dXdb9aspeI8k8fiI+fsY=rqGqVepae9pg0db9vqaiVgFr0xfr=xfr=xc9adbaqaaeGacaGaaiaabeqaaeqabiWaaaGcbaWenfgDOvwBHrxAJfwnHbqeg0uy0HwzTfgDPnwy1aaceaGae8xdX7eaaa@3761@)) which condition on the degree sequence (by virtue of fixing the degree of each node) and on the weight matrix *ω *(by construction of the chain).

As in all MCMC approaches it is important to run the algorithm for a sufficiently long period to remove dependence on the initial configuration and to reach the stationary distribution of the Markov Process (the *burn-in period*). After that the chain produces highly correlated configurations so configurations are sampled only after a sufficiently large number of steps in the chain (this is referred to as the *thinning-out interval*) [35,36]. Choice of the length for burn-in and thinning-out intervals require experimentation and/or fine-tuning. In GOcardShuffle the default parameter for the burn-in period is 100 × *M *steps, while the thinning-out interval has a length of 10 × *M *steps.

#### Generalizations

In the discussion we have thus far assumed that each protein has only one annotation. Two additional factors are straightforwardly included in GOcardShuffle:

**Multiple annotations: **For many proteins we have more than one annotation. This can be due to a protein being found in more than one cellular component; being involved in more than one biological process; or having more than one molecular function; or any combination of the above.

**Multiple annotation categories: **Above we have chosen to group proteins together if they have identical annotations. Thus *ν*_*x *_is the number of proteins with the same annotation *x*; this means that they all have the same annotation regarding function, process and component. If each category has 30 annotations then we need to consider 27,000 unique annotations and approximately 3.6 × 10^8 ^different combinations *x*, *y *∈ *γ*, most of which will be zero.

*Multiple annotation *can be easily incorporated into GOcardShuffle. If a protein has annotations *x*_1 _and *x*_2_, then its probability of interacting with a protein with annotation *y *is given by

ω(x1,x2)y=12(ωx1y+ωx2y).
 MathType@MTEF@5@5@+=feaafiart1ev1aaatCvAUfKttLearuWrP9MDH5MBPbIqV92AaeXatLxBI9gBaebbnrfifHhDYfgasaacPC6xNi=xI8qiVKYPFjYdHaVhbbf9v8qqaqFr0xc9vqFj0dXdbba91qpepeI8k8fiI+fsY=rqGqVepae9pg0db9vqaiVgFr0xfr=xfr=xc9adbaqaaeGacaGaaiaabeqaaeqabiWaaaGcbaacciGae8xYdC3aaSbaaSqaaiabcIcaOiabdIha4naaBaaameaacqaIXaqmaeqaaSGaeiilaWIaemiEaG3aaSbaaWqaaiabikdaYaqabaWccqGGPaqkcqWG5bqEaeqaaOGaeyypa0tcfa4aaSaaaeaacqaIXaqmaeaacqaIYaGmaaGccqGGOaakcqWFjpWDdaWgaaWcbaGaemiEaG3aaSbaaWqaaiabigdaXaqabaWccqWG5bqEaeqaaOGaey4kaSIae8xYdC3aaSbaaSqaaiabdIha4naaBaaameaacqaIYaGmaeqaaSGaemyEaKhabeaakiabcMcaPiabc6caUaaa@4ABA@

This assumes that annotations *x*_1 _and *x*_2 _are equally important in describing the biological characteristics of protein *x*. If, for example, *x*_1 _is more relevant then we would have to replace Eqn. (10) by ω(x1,x2)y=12(w1ωx1y+(1−w1)ωx2y)
 MathType@MTEF@5@5@+=feaafiart1ev1aaatCvAUfKttLearuWrP9MDH5MBPbIqV92AaeXatLxBI9gBaebbnrfifHhDYfgasaacPC6xNi=xH8viVGI8Gi=hEeeu0xXdbba9frFj0xb9qqpG0dXdb9aspeI8k8fiI+fsY=rqGqVepae9pg0db9vqaiVgFr0xfr=xfr=xc9adbaqaaeGacaGaaiaabeqaaeqabiWaaaGcbaacciGae8xYdC3aaSbaaSqaaiabcIcaOiabdIha4naaBaaameaacqaIXaqmaeqaaSGaeiilaWIaemiEaG3aaSbaaWqaaiabikdaYaqabaWccqGGPaqkcqWG5bqEaeqaaOGaeyypa0tcfa4aaSaaaeaacqaIXaqmaeaacqaIYaGmaaGccqGGOaakcqWG3bWDdaWgaaWcbaGaeGymaedabeaakiab=L8a3naaBaaaleaacqWG4baEdaWgaaadbaGaeGymaedabeaaliabdMha5bqabaGccqGHRaWkcqGGOaakcqaIXaqmcqGHsislcqWG3bWDdaWgaaWcbaGaeGymaedabeaakiabcMcaPiab=L8a3naaBaaaleaacqWG4baEdaWgaaadbaGaeGOmaidabeaaliabdMha5bqabaGccqGGPaqkaaa@5251@. In most cases, however, present information will not be sufficient to introduce reliable weightings of multiple annotations for each protein.

Therefore we continue along the more parsimonious route of attaching equal weight to all multiple annotations and write more generally, for proteins with annotations given by **x **= (*x*_1_, *x*_2_,...,*x*_*α*_) and **y **= (*y*_1_, *y*_2_,...,*y*_*β*_), respectively, we have

ωxy=1αβ(∑i=1α∑j=1βωxiyj)
 MathType@MTEF@5@5@+=feaafiart1ev1aaatCvAUfKttLearuWrP9MDH5MBPbIqV92AaeXatLxBI9gBaebbnrfifHhDYfgasaacPC6xNi=xI8qiVKYPFjYdHaVhbbf9v8qqaqFr0xc9vqFj0dXdbba91qpepeI8k8fiI+fsY=rqGqVepae9pg0db9vqaiVgFr0xfr=xfr=xc9adbaqaaeGacaGaaiaabeqaaeqabiWaaaGcbaacciGae8xYdC3aaSbaaSqaaGqabiab+Hha4jab+Lha5bqabaGccqGH9aqpjuaGdaWcaaqaaiabigdaXaqaaiab=f7aHjab=j7aIbaakmaabmaabaWaaabCaeaadaaeWbqaaiab=L8a3naaBaaaleaacqWG4baEdaWgaaadbaGaemyAaKgabeaaliabdMha5naaBaaameaacqWGQbGAaeqaaaWcbeaaaeaacqWGQbGAcqGH9aqpcqaIXaqmaeaacqWFYoGya0GaeyyeIuoaaSqaaiabdMgaPjabg2da9iabigdaXaqaaiab=f7aHbqdcqGHris5aaGccaGLOaGaayzkaaaaaa@4EE9@

With Eqn. (11) the normalization of the edge probabilities *ω*_*x*,*y *_is trivially maintained. Multiple annotations are therefore straightforwardly and parsimoniously dealt with. Once annotations become very reliable and detailed it will, however, be possible to introduce weightings on different annotations. Alternatively to Eqn. (11) we may determine that a combination of annotations **x **= (*x*_1_, *x*_2_,...,*x*_*α*_) defines a new annotation. This could be advantageous if proteins that have more than one function, *i.e. *annotation **x***' *tend to interact predominantly with proteins that have a different annotation *x" *(or set of annotations **x***"*). Clearly in such an event the simple ansatz given by Eqn. (11) may give rise to interactions among proteins that would never interact in real life. Combining annotations into a new single annotation is possible by preprocessing the annotation input-data prior to using GOcardShuffle. Given the present state of the data (both PIN and annotations) we believe that using the approach given by Eqn. (11) puts less emphasis on potentially erroneous data; in the future, however, it will be possible to go beyond this approach by considering dependencies among sets of annotations.

Dealing with the potentially very large number of different annotations requires more careful consideration. In addition to the computational challenges of dealing with very large matrices, *ω *= (*ω*_*xy*_), taking annotations as "true" could be problematic as it may severely limit the size of the network ensemble that is defined through the stationary distribution of the Markov Chain defined by GOcardShuffle as most entries in *ω *will be zero. An additional problem is that GO annotation is only approximate and when protein-interaction data has been used to annotate proteins *in silico *errors in either the interaction data or GO annotations may be propagated. A pragmatic if approximate solution is to divide the annotations into the three different categories: molecular function; biological process; and cellular component. We thus define 3 different matrices, one for each category

*ω*^**k**^   for *k *= 1, 2, 3   or *k *= *F*, *P*, *C*

n the R-implementation of GOcardShuffle the user has the choice of using individual matrices, *ω*^**k**^, a compound matrix, *ω *(as discussed above), or an approximation to *ω *given by

ω˜=ω1⊗ω2⊗ω3
 MathType@MTEF@5@5@+=feaafiart1ev1aaatCvAUfKttLearuWrP9MDH5MBPbIqV92AaeXatLxBI9gBaebbnrfifHhDYfgasaacPC6xNi=xI8qiVKYPFjYdHaVhbbf9v8qqaqFr0xc9vqFj0dXdbba91qpepeI8k8fiI+fsY=rqGqVepae9pg0db9vqaiVgFr0xfr=xfr=xc9adbaqaaeGacaGaaiaabeqaaeqabiWaaaGcbaacciGaf8xYdCNbaGaacqGH9aqpcqWFjpWDdaahaaWcbeqaaiabigdaXaaakiabgEPielab=L8a3naaCaaaleqabaGaeGOmaidaaOGaey4LIqSae8xYdC3aaWbaaSqabeaacqaIZaWmaaaaaa@3BE5@

(or any combination of pairs of annotations, ω˜
 MathType@MTEF@5@5@+=feaafiart1ev1aaatCvAUfKttLearuWrP9MDH5MBPbIqV92AaeXatLxBI9gBaebbnrfifHhDYfgasaacPC6xNi=xH8viVGI8Gi=hEeeu0xXdbba9frFj0xb9qqpG0dXdb9aspeI8k8fiI+fsY=rqGqVepae9pg0db9vqaiVgFr0xfr=xfr=xc9adbaqaaeGacaGaaiaabeqaaeqabiWaaaGcbaacciGaf8xYdCNbaGaaaaa@2DB6@ = *ω*^**i **^⊗ *ω*^**j **^for *i*, ≠ *j *∈ 1, 2, 3). In Eqn. (12), ⊗ denotes the standard tensor product [37] of the weight matrices. This has also numerical and computational advantages as we only have to store three (or two) small matrices (typically we use approximately 30 annotations per category) rather than one very large matrix. Eqn. (12) will for real networks only be approximate if the different GO categories are themselves correlated (which we know to be the case for yeast and other organisms for which extensive GO annotation data has been assembled) and it will be necessary to test whether this approximation is reasonable (in the data presented here we found acceptably small differences between the true and approximate weights). Nevertheless, even if only the approximation is used, any systematic differences between classical rewiring approaches and the network instances created by GOcardShuffle will highlight confounding factors which ought to be included in the construction of network confidence measures.

The GOcardShuffle algorithm can be summarized as follows

GOcardShuffle

   Generate set of stubs from true network

   **while **free stubs **do**

      Choose two stubs uniformly from those remaining and create an edge between them

   **end while**

   **for ***i *= 0 to *λ ***do**

      Choose two edges *a *and *b *in current network at random, uniformly

      Calculate *p *using Eqn. (6).

      Generate random value 0 <*r *< 1

      **if ***r *<*p *or *p *> 1 **then**

         Cross over *a *and *b *in network

      **end if**

   **end for**

The chain is sampled at intervals of *λ*_1 _steps, after a burn-in period of *λ*_0 _steps. For GOcardShuffle the default values are

*λ*_0 _= 100 × *M*   and   *λ*_1 _= 10 × *M*,

(*M *is again the number of edges in the network). If *L *conditionally rewired network instances are required then *λ *= 100 × *M *+ 10 × *M *× *L*.

### Motifs

In this paper only network motifs containing 4 nodes were considered. In an undirected network there are only six possible non-isomorphic configurations of edges between 4 nodes, these are shown at the bottom of figure 2. Motif spectra where calculated according to Milo *et al*. (see [14,15]). The statistical significance of a motif is assessed using the *Z*-score, which is defined as

Z=n−〈n〉σn
 MathType@MTEF@5@5@+=feaafiart1ev1aaatCvAUfKttLearuWrP9MDH5MBPbIqV92AaeXatLxBI9gBaebbnrfifHhDYfgasaacPC6xNi=xI8qiVKYPFjYdHaVhbbf9v8qqaqFr0xc9vqFj0dXdbba91qpepeI8k8fiI+fsY=rqGqVepae9pg0db9vqaiVgFr0xfr=xfr=xc9adbaqaaeGacaGaaiaabeqaaeqabiWaaaGcbaGaemOwaOLaeyypa0tcfa4aaSaaaeaacqWGUbGBcqGHsislcqGHPms4cqWGUbGBcqGHQms8aeaaiiGacqWFdpWCdaWgaaqaaiabd6gaUbqabaaaaaaa@398C@

where *n *is the number of times the motif is found in the true network, ⟨*n*⟩ is the average number of times the same motif is found in the rewired networks, and *σ*_*n *_is the standard deviation of motif counts in the rewired network. reduce the amount combinations that need to be considered, only nodes within a path of length 1 from the current node are considered for the choice of the second node, and nodes within a path of length 1 from the first or second node for the third node.

### Implementation

The methods described above were implemented in Python, as well as for the R statistical environment [38] (computationally intensive routines were implemented in C); R was also used for all statistical analyses. The source code for the GOcardShuffle algorithm is available from our website [22].

## Authors' contributions

TT and MPHS jointly designed the study, developed the approach and wrote the manuscript. The algorithms were implemented and applied to the Yeast protein interaction data by TT. All authors read and approved the final manuscript.

## Supplementary Material

Additional file 1 **Supplementary Material**. Discussion of statistical properties of GOCardShuffle. Click here for file
