# Supplementary material for: Generating confidence intervals on biological networks
Source: BMC Bioinformatics. 2007 Nov 30;8:467. doi: 10.1186/1471-2105-8-467 (PMC2241843; doi:10.1186/1471-2105-8-467)
Supplement: Additional file 1 — Supplementary Material. Discussion of statistical properties of GOCardShuffle. [file 1471-2105-8-467-S1.pdf]

Supplementary Material: Generating Confidence Intervals on Biological Networks

T. Thorne & M.P.H. Stumpf

April 5, 2007

1 Effect of Dataset choice on GOCardShuffle results

The poor quality of different protein interaction datasets has attracted great interest in the literature (see *e.g.* [2,5,9]). We have applied the `GOCardShuffle` algorithm to a range of datasets (see figure 1). In each case we find that the confidence intervals obtained without and with conditioning on available GO annotation are significantly different. For all datasets we also observe that the `GOCardShuffle` confidence intervals for Kendall's τ rank correlation coefficient overlap the observed correlation in each network dataset but for the literature curated (denoted by LC in figure 1) data of Reguly *et al.* [5] and the DIP CORE dataset [1,2,10] we find that the expression levels of interacting proteins are more similar than would be expected to be the case by chance. We note, however, that on this level, in addition to dataset choice also the correlation measure becomes important and for a given dataset `GOCardShuffle` Null distributions for one correlation coefficient may overlap the observed network statistic while for a different correlation coefficient this is no longer the case. In such a case evidence for similarity of the properties of interacting proteins would probably have to be considered marginal.

In summary, however, this shows that there is a need for statistical methods that condition on the available data.

2 Illustration of GOCardShuffle

The algorithm outlined in the manuscript and implemented in the accompanying software (and the `NetZ` package; www.imperial.ac.uk/theoreticalgenomics/data-software).

In figure 2 we show the distribution of edge numbers connecting nodes with certain funcional categories resulting from 400 conventionally rewired networks(black histograms) and from 400 networks where rewiring was conditioned on GO functions using `GOCardShuffle`. Although this is only a small selection of within and between category edges these are representative for the remaining cases. In each case we find that the histogram obtained using `GOCardShuffle` overlaps the observed value. For some instances, notably for between-category edges where one protein has no known annotation, unconditional rewiring results in a distribution which covers the observed number of between-category edges in the original network. In particular unconditional rewiring underestimates the relative prevalence of within-category connections [3,7].

For a single annotation matrix ω the number of edges between proteins beloning to categories i and j, m_{ij} under unconditional resampling has the expectation value

$$E[m_{xy}] = M\frac{N_x}{N}\frac{N_y}{N} = M\frac{N_xN_y}{N^2} \tag{1}$$

where M is the total number of edges in the network, N_x is the number of nodes/proteins beloning to category i and N is the total number of nodes in the network; its variance is given by the standard multinomial variance

$$\mathrm{Var}[m_{xy}] = M\frac{N_xN_y}{N^2}\left(1 - \frac{N_xN_y}{N^2}\right) \tag{2}$$

Since edges are sampled uniformly the Markov Chain generated by Eqns. (5) to (8) in the manuscript will converge to the desired stationary distribution for the within and between category connection pattern [6,8].

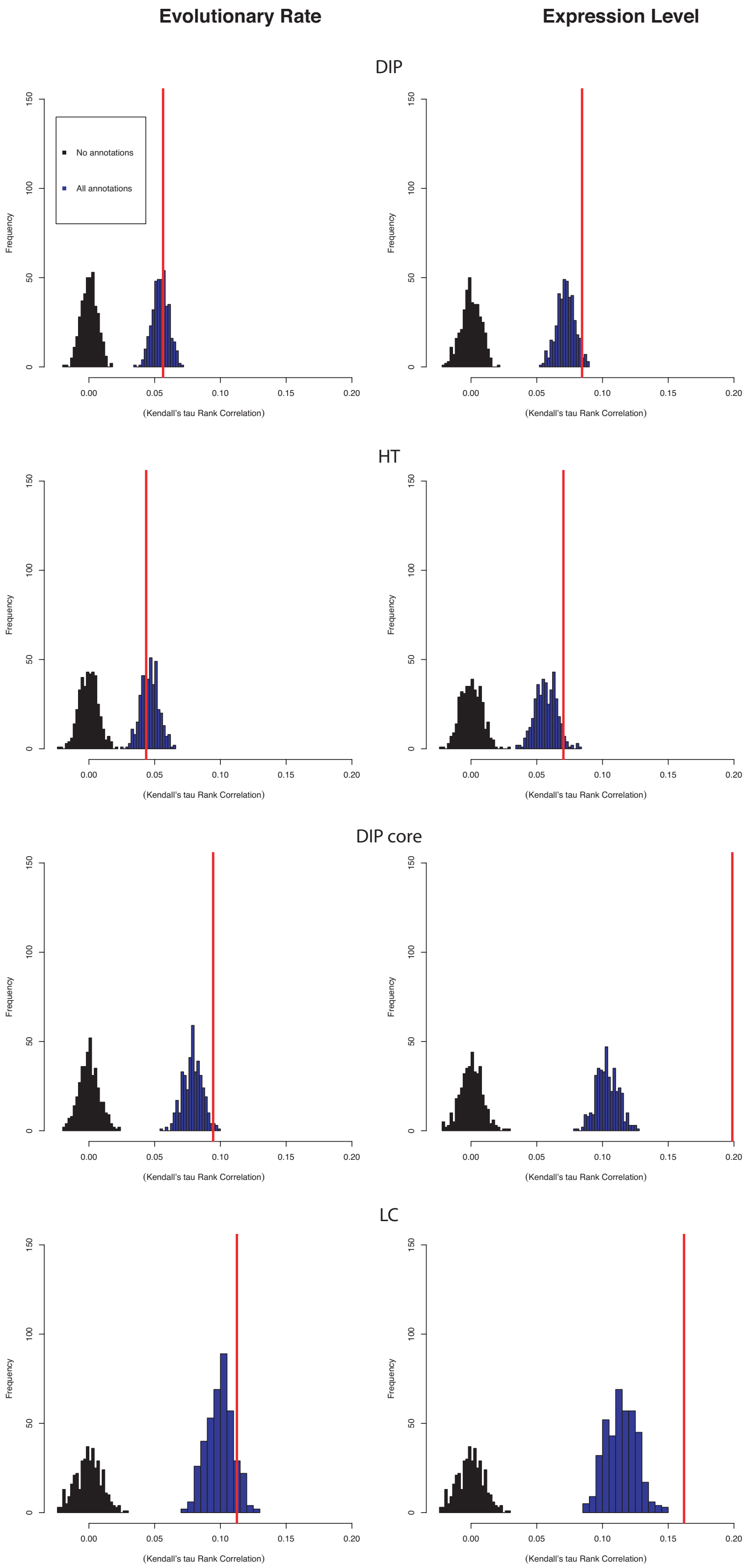

Figure 1: Histograms of Kendall's τ rank correlation coefficients obtained from normal rewiring (black) and for `GOCardShuffle` (blue). Also shown are the observed values (vertical red lines) of τ in the four datasets. The LC and HT datasets are the literature curated and pooled high-thoughput datasets of Reguly *et al.* [5], the DIP and DIP core datasets are the complete and high-confidence interactions deposited in the database of interacting proteins [1, 10].

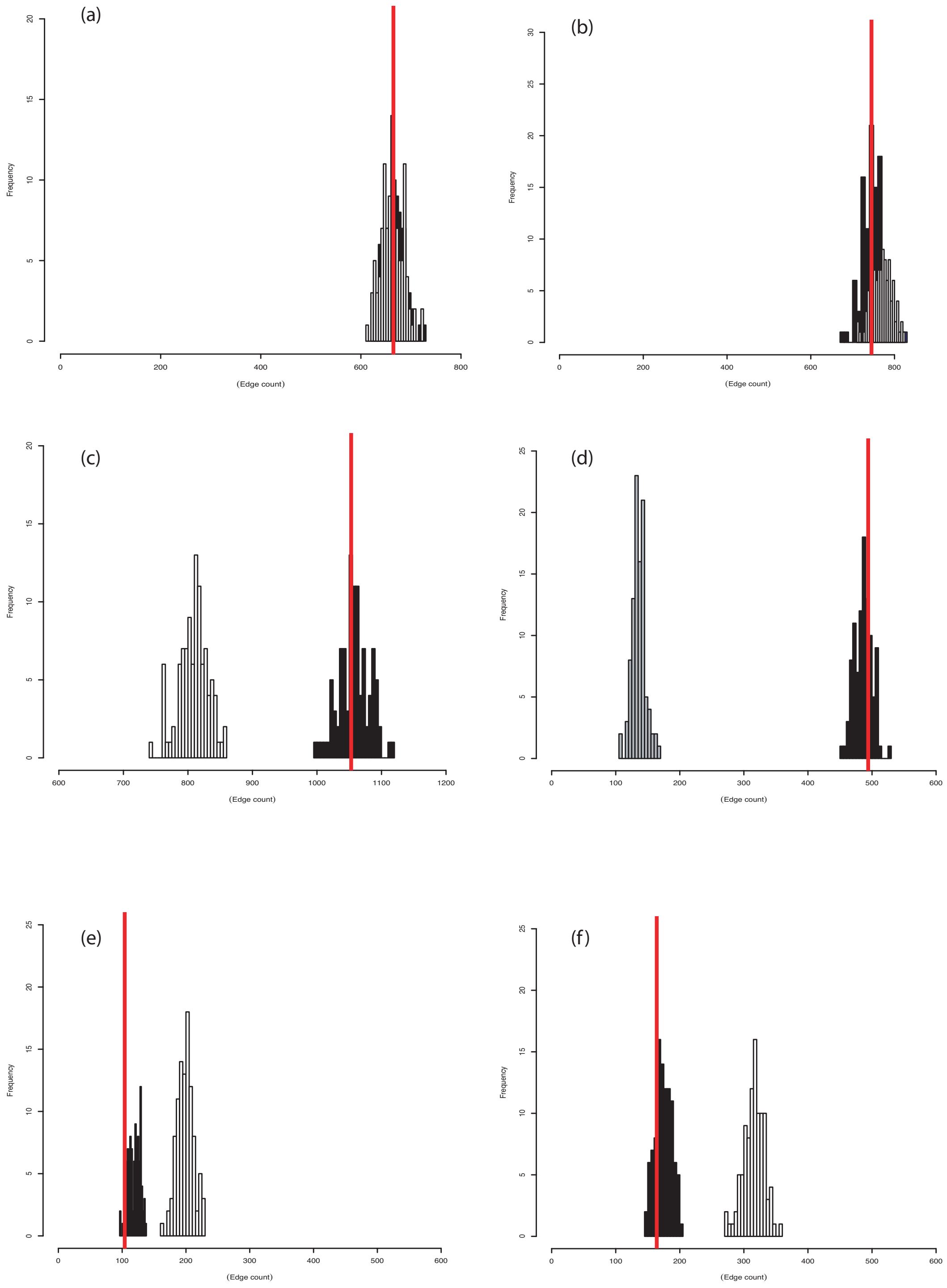

Figure 2: Number of edges observed in the real network connecting nodes with specific functional GO categories (red line) and histograms of these numbers obtained from 400 independent network instances using unconditional rewiring (white) and conditional rewiring using `GOcardShuffle` (black), respectively. The different panels show edges connecting proteins belonging to the following functional categories: (a) "protein-function unknown" — "RNA binding"; (b) "protein-function unknown" — "protein binding"; (c) "protein-function unknown" — "protein-function unknown"; (d) "protein-function unknown" — "RNA binding"; (e) "RNA binding" — "protein binding"; (f) "transcription regulator activity" — "RNA binding".

The variability of the number of edges between and with categories, cannot be assessed analytically for all

but regular random graphs (*i.e.* graphs where each node has the same fixed degree but edges are distributed at random) as there is a delicate interplay between (i) the variance induced by the Metropolis sampler, and (ii) the variation induced by heterogenous node degrees. The results shown in figure 2, together with the observation that the histograms for within and between-category edge numbers always include the observed value, fills us with confidence, that `GOCardShuffle` does indeed preserve the hallmarks of the original network data.

References

[1] dip.doe-mbi.ucla.edu.

[2] Joel S Bader, Amitabha Chaudhuri, Jonathan M Rothberg, and John Chant. Gaining confidence in high-throughput protein interaction networks. *Nat Biotechnol*, 22(1):78–85, Jan 2004.

[3] Julien Gagneur, Roland Krause, Tewis Bouwmeester, and Georg Casari. Modular decomposition of protein-protein interaction networks. *Genome Biology*, 5(8):R57, 2004.

[4] N. Metropolis, A.W. Rosenbluth, M.N. Rosenbluth, A.H. Teller, and E. Teller. Equation of state calculations by fast computing machines. *J.Chem.Phys.*, 21:1087–1092, 1953.

[5] T. Reguly, A. Breitkreutz, L. Boucher, B.J. Breitkreutz, G.C. Hon, C.L. Myers, A. Parsons, H. Friesen, A. Oughtred, R. amd Tong, C. Stark, Y. Ho, D. Botstein, B. Andrews, C. Boone, O.G. Troyanskya, T. Ideker, K. Dolinski, N.N. Batada, and M. Tyers. Comprehensive curation and analysis of global interaction networks in saccharomyces cerevisiae. *J.Biol.*, 5:11, 2006.

[6] Brian D. Ripley. *Stochastic Simulation.* Wiley, 1987.

[7] Alexander W Rives and Timothy Galitski. Modular organization of cellular networks. *Proc. Natl. Acad. Sci. U S A*, 100(3):1128–33, Feb 2003.

[8] C.P. Robert and G. Casella. *Monte Carlo Statistical Methods.* Springer, 2nd edition, 2004.

[9] Christian von Mering, Roland Krause, Berend Snel, Michael Cornell, Stephen G Oliver, Stanley Fields, and Peer Bork. Comparative assessment of large-scale data sets of protein-protein interactions. *Nature*, 417(6887):399–403, May 2002.

[10] I. Xenarios, D. Rice, L. Salwinski, M. Baron, E. Marcotte, , and D. Eisenberg. Dip: the database of interacting proteins. *Nucl.Acid.Res.*, 28:289–291, 2000.
